# Supplementary material for: Obesity and disease severity magnify disturbed microbiome-immune interactions in asthma patients
Source: Nat Commun. 2019 Dec 13;10:5711. doi: 10.1038/s41467-019-13751-9 (PMC6911092; doi:10.1038/s41467-019-13751-9)
Supplement: Supplementary file 1 — Supplementary Information [file 41467_2019_13751_MOESM1_ESM.pdf]

Supplementary Information

Obesity and Disease Severity Magnify Disturbed Microbiome-Immune Interactions in Asthma Patients

Michalovich et al.

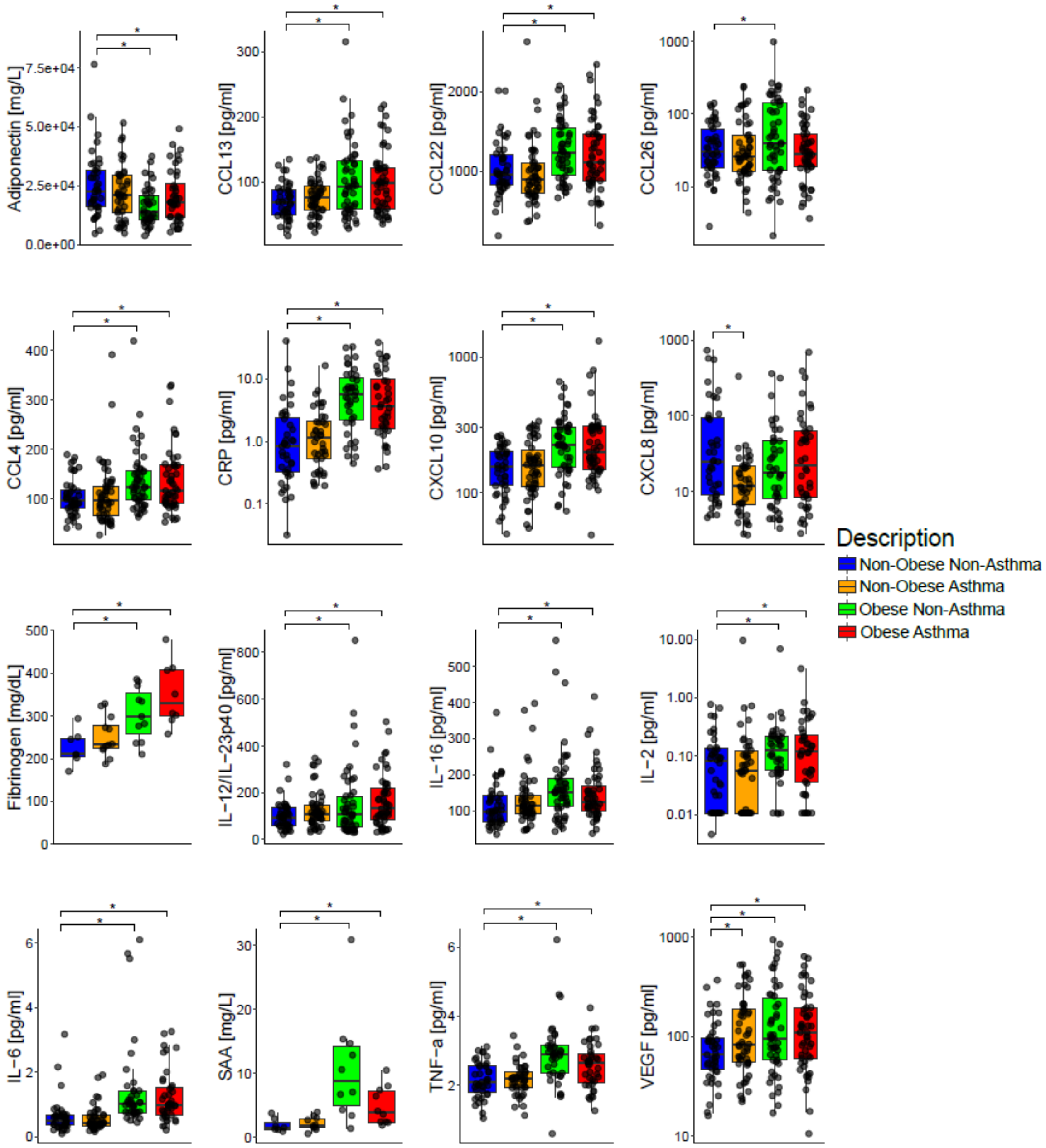

Supplementary Figure 1. Serum biomarker levels.

Data were analyzed by one-way ANOVA (correcting for gender) and Tukey's multiple comparison post hoc test and significant differences ($p < 0.05$) are labeled with asterisks for obese non-asthma (n=50), obese asthma (n=50) and non-obese asthma (n=52) patients relative to the non-obese non-asthmatic group (n=47) are reported. Box plots show median and whiskers represent 10-90 percentiles.

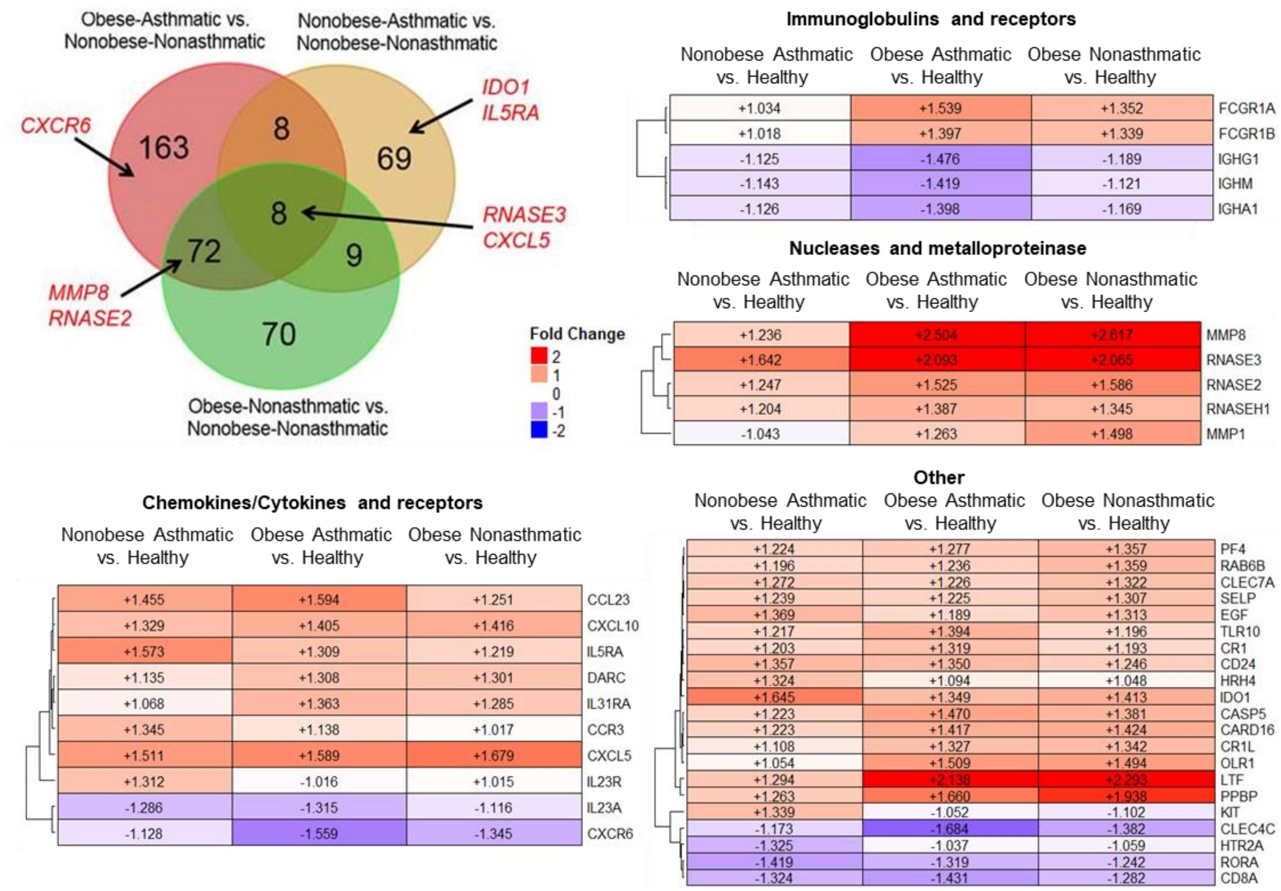

Supplementary Figure 2. DEGs from whole blood microarray analysis.

Venn diagram illustrates the number and overlap for differentially expressed genes (DEGs) for obese non-asthma (n=51), obese asthma (n=50) and non-obese asthma (n=53) patients compared to the non-obese non-asthmatic group (n=48). Expanded heat maps illustrate immunologically relevant DEGs from whole blood microarrays.

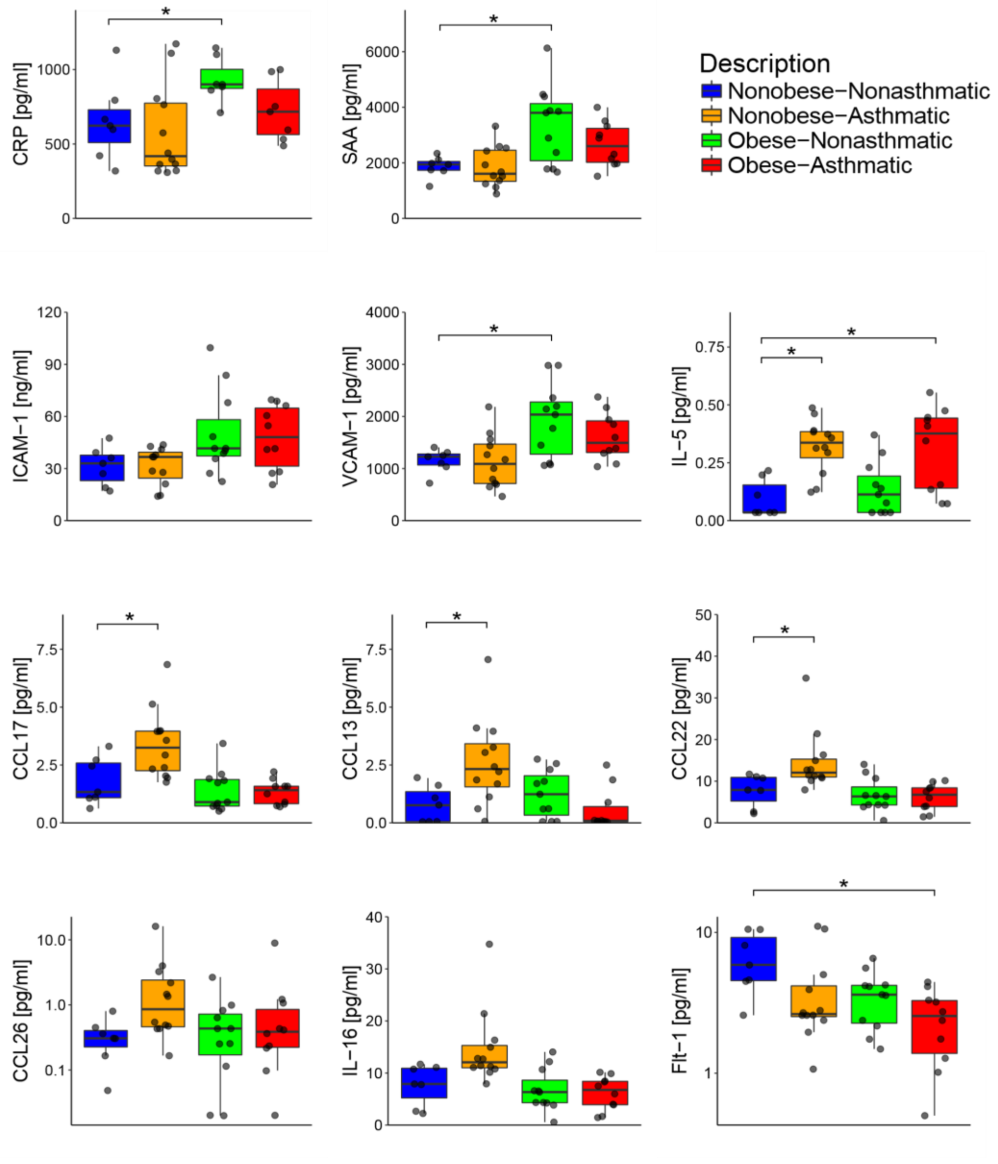

Supplementary Figure 3. BAL biomarker levels.

Data were analyzed by one-way ANOVA (correcting for gender) and Tukey’s multiple comparison post hoc test and significant differences ($p < 0.05$) are labeled with asterisks for obese non-asthma (n=11), obese asthma (n=10) and non-obese asthma (n=12) patients relative to the non-obese non-asthmatic group (n=7) are reported. Box plots show median and whiskers represent 10-90 percentiles.

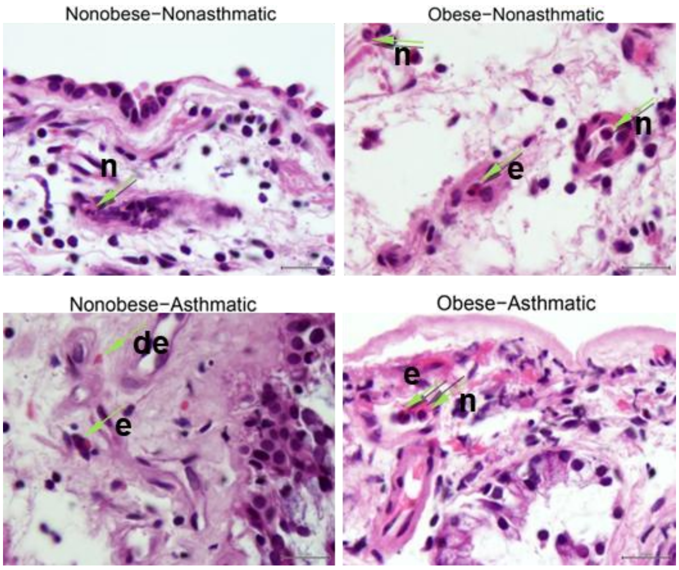

Supplementary Figure 4. Biopsy histology.

H&E staining of selected lung biopsies illustrating the presence of eosinophils (e), degranulated eosinophils (de) and neutrophils (n).

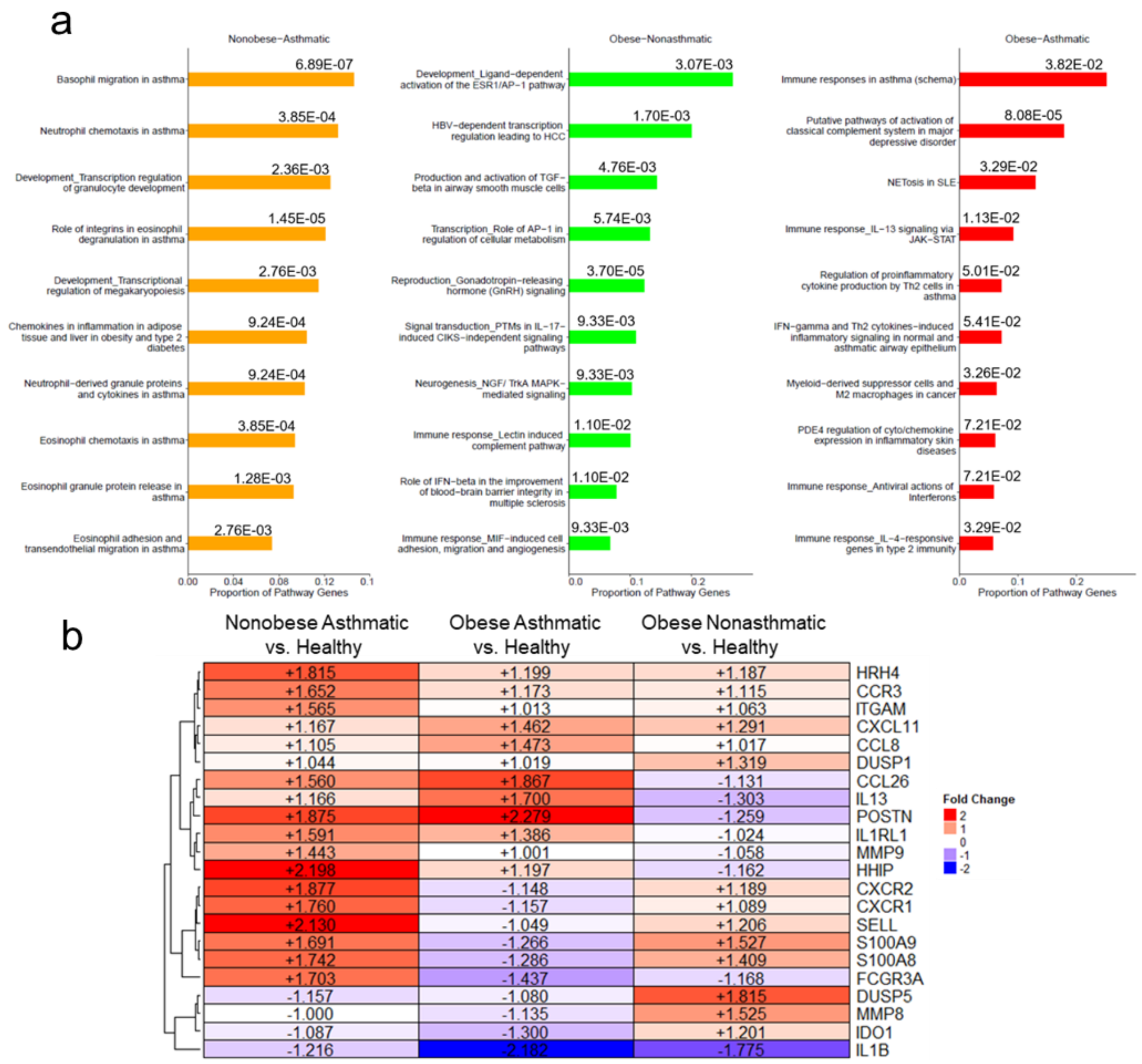

Supplementary Figure 5. Pathway enrichments and DEGs from bronchial biopsy RNA sequencing analysis.

a The top 10 gene ontology pathway enrichments in lung biopsies for each group (Non-obese asthma (n=12); Obese non-asthma (n=11); Obese asthma (n=10)) compared to non-obese and non-asthma volunteers (n=8) are illustrated using both the proportion of the pathway or ontology gene set that overlapped with the differentially expressed genes in that comparison and the FDR-corrected p-values as a label next to the bar. **b** Expanded heatmaps of

immunologically relevant differentially expressed genes (DEGs) from biopsy RNA-seq for non-obese asthma (n=12), obese non-asthma (n=11) and obese asthma (n=10) patients compared to non-obese and non-asthma volunteers (n=8).

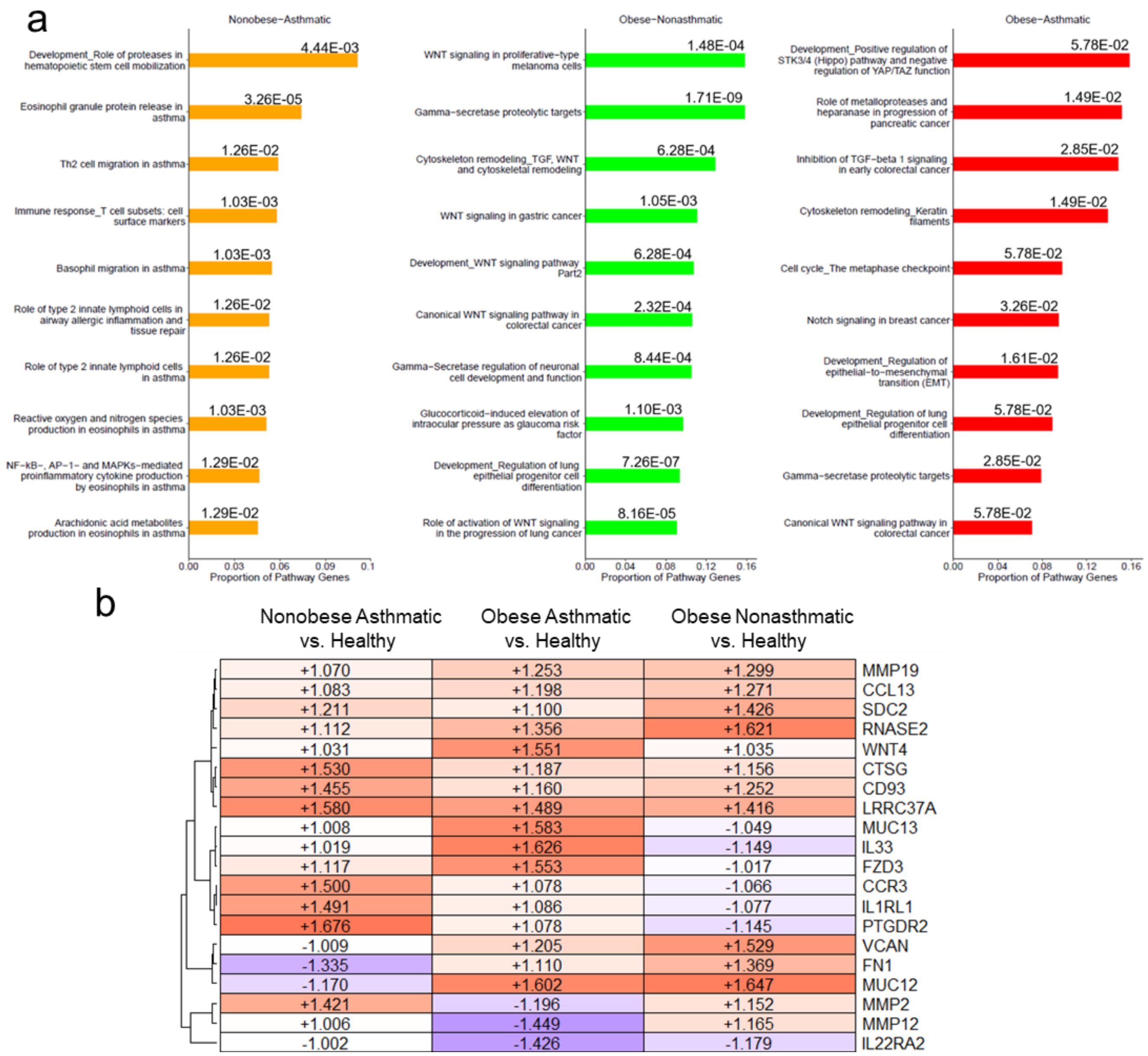

Supplementary Figure 6. Pathway enrichments and DEGs from BAL RNA sequencing analysis.

a The top 10 gene ontology pathway enrichments in BALs for each group (Non-obese asthma (n=12); Obese non-asthma (n=11); Obese asthma (n=10)) compared to non-obese and non-asthma volunteers (n=8) are illustrated using both the proportion of the pathway or ontology gene set that overlapped with the differentially expressed genes in that comparison and the

FDR-corrected p-values as a label next to the bar. **b** Expanded heatmaps of immunologically relevant differentially expressed genes (DEGs) from BAL RNA-seq for non-obese asthma (n=12), obese non-asthma (n=11) and obese asthma (n=10) patients compared to non-obese and non-asthma volunteers (n=8).

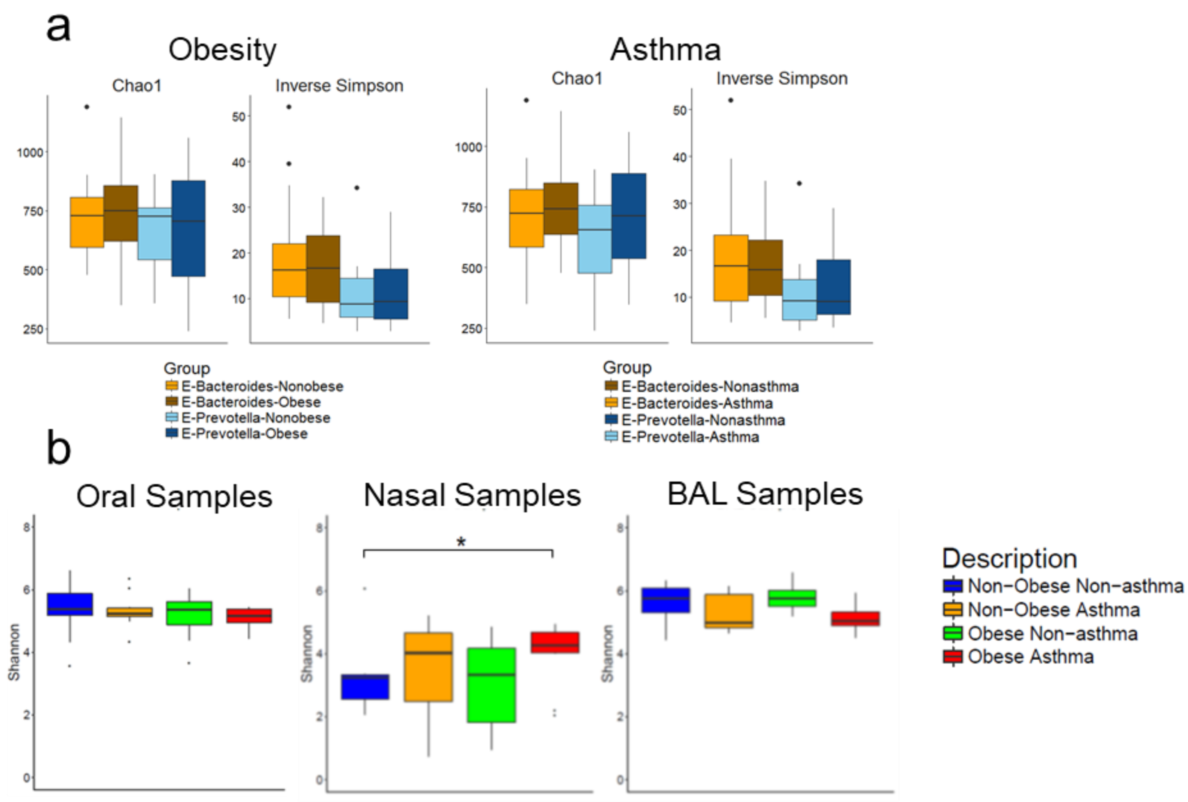

Supplementary Figure 7. Microbiota diversity.

a Gut microbiome alpha diversity, corrected for enterotype, are not significantly different for non-obese asthma (n=53), obese non-asthma (n=51), or obese asthma (n=50) relative to the non-obese non-asthmatic group (n=48). **b** No statistically significant differences are observed in alpha diversity measures between non-obese asthma (n=12), obese non-asthma (n=11), obese asthma (n=10) or non-obese non-asthma (n=8) for BAL or oral samples, while the obese asthma nasal Shannon diversity was increased relative to healthy volunteers (one-way ANOVA), labeled with an asterisk. Box plots show median and whiskers represent 10-90 percentiles.

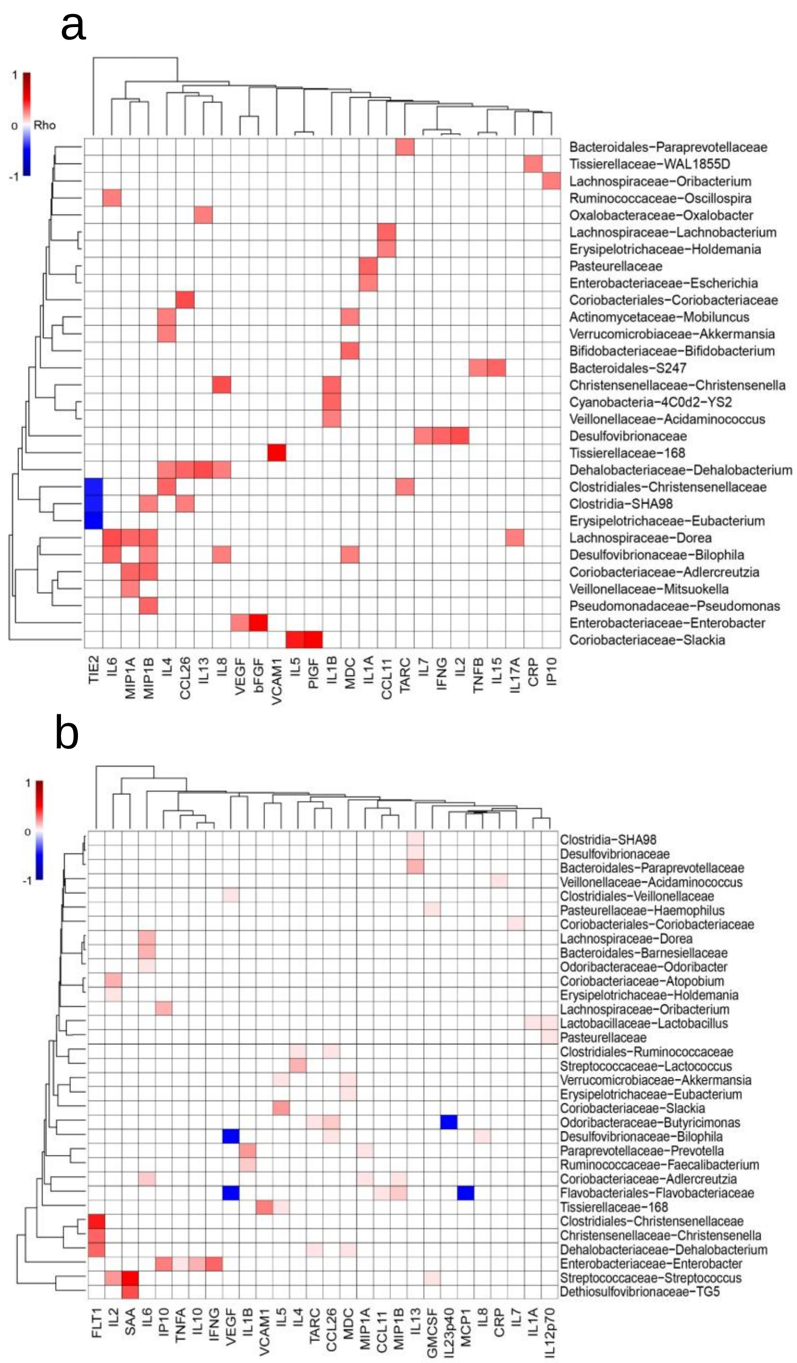

Supplementary Figure 8. Associations between serum biomarkers and microbes within the gut.

The significant correlations (Pearson test, $p<0.01$) between serum biomarkers and the relative abundance of gut microbes in **a** obese (n=101) and **b** asthma (n=53) patients are illustrated.

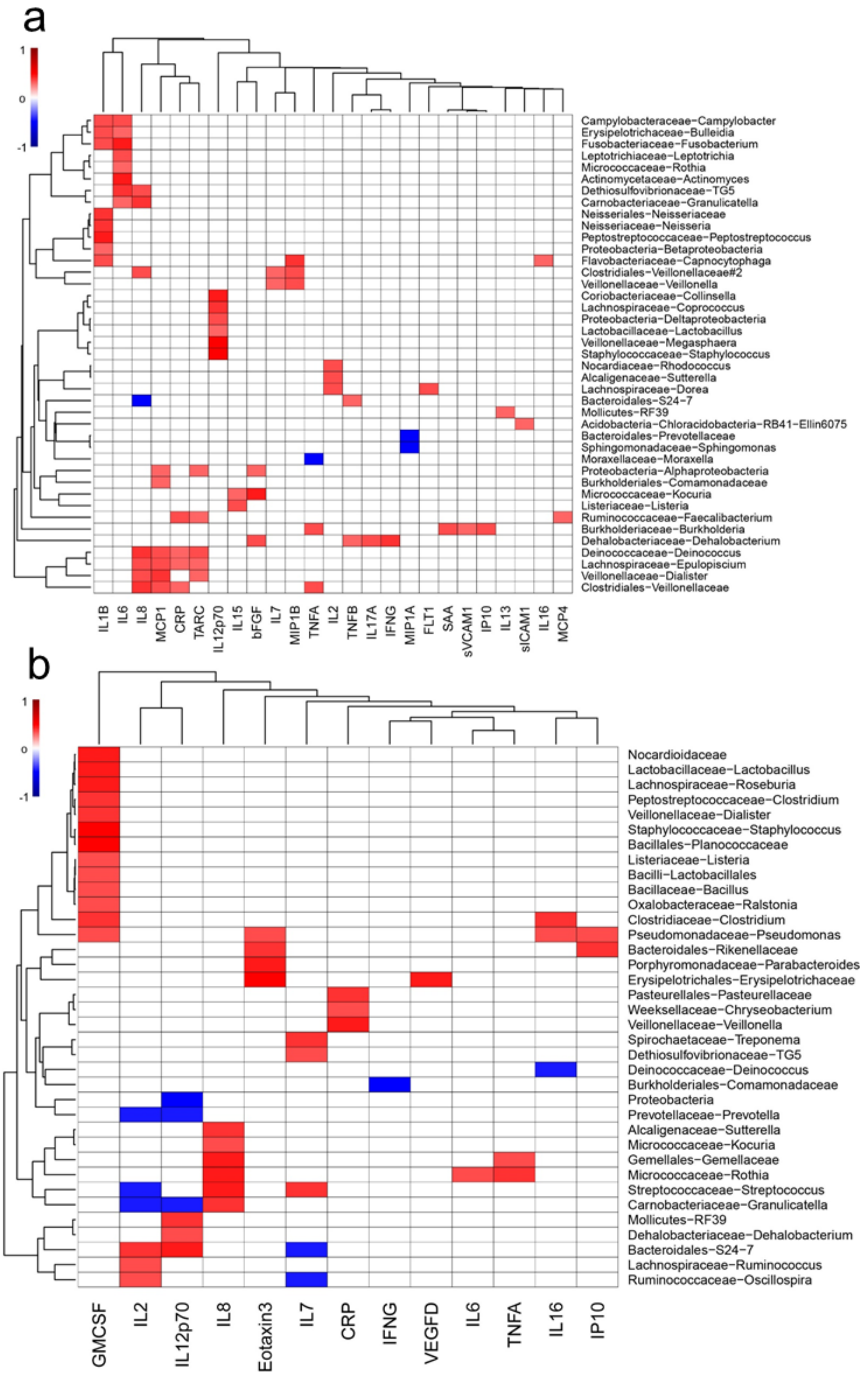

Supplementary Figure 9. Associations between BAL biomarkers and microbes within the lung.

The significant correlations (Pearson, $p<0.01$) between BAL biomarkers and the relative abundance of BAL microbes in **a** obese (n=21) and **b** asthma (n=12) patients are illustrated.

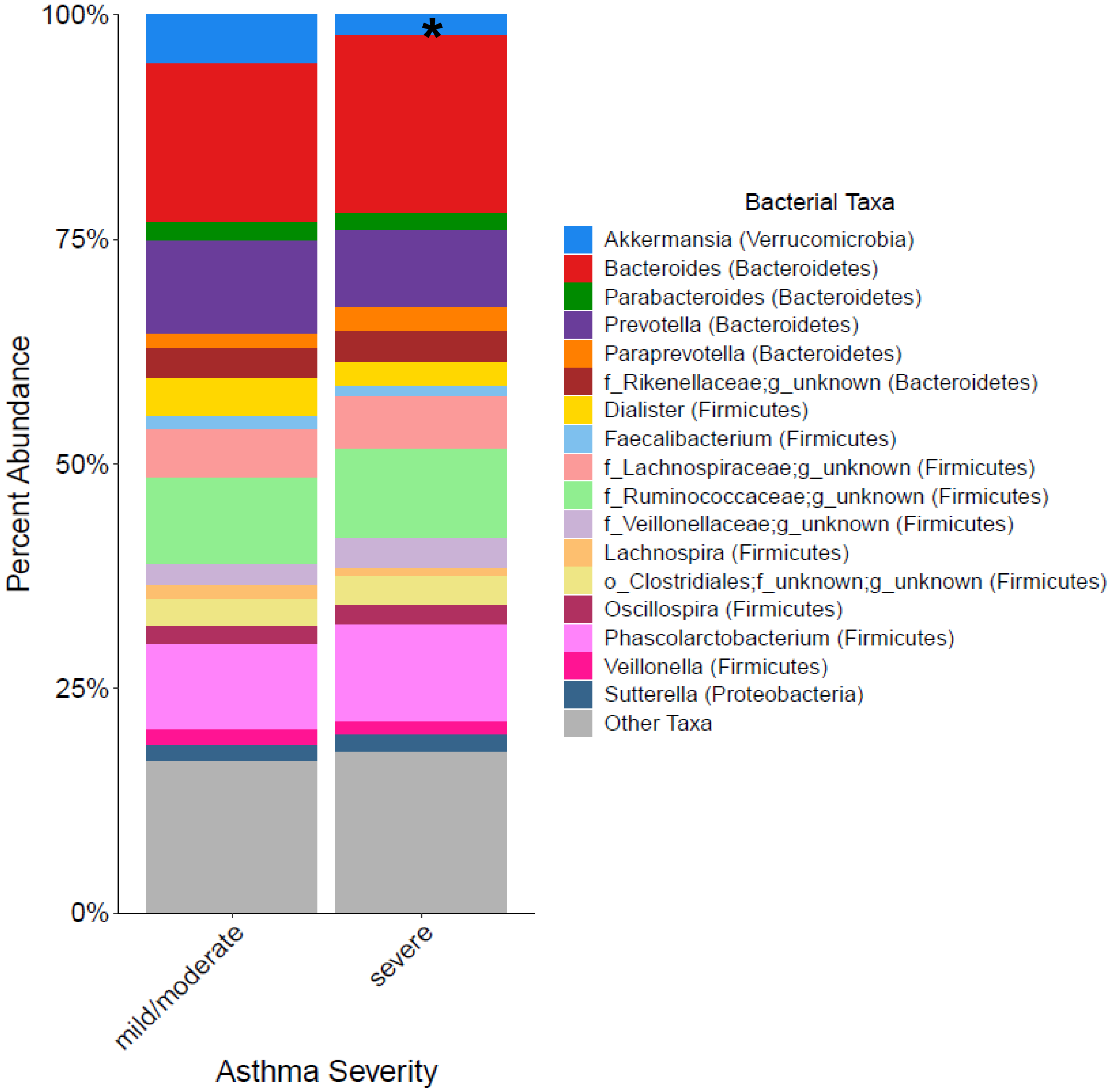

Supplementary Figure 10. *A. muciniphila* is negatively correlated with severe asthma.

Relative abundances at the Genus taxonomic level within the fecal samples of asthma patients with mild/moderate (n=53) or severe (n=41) disease are illustrated (asterisk denotes a P < 0.05, ANOVA with post hoc Tukey-kramer test).

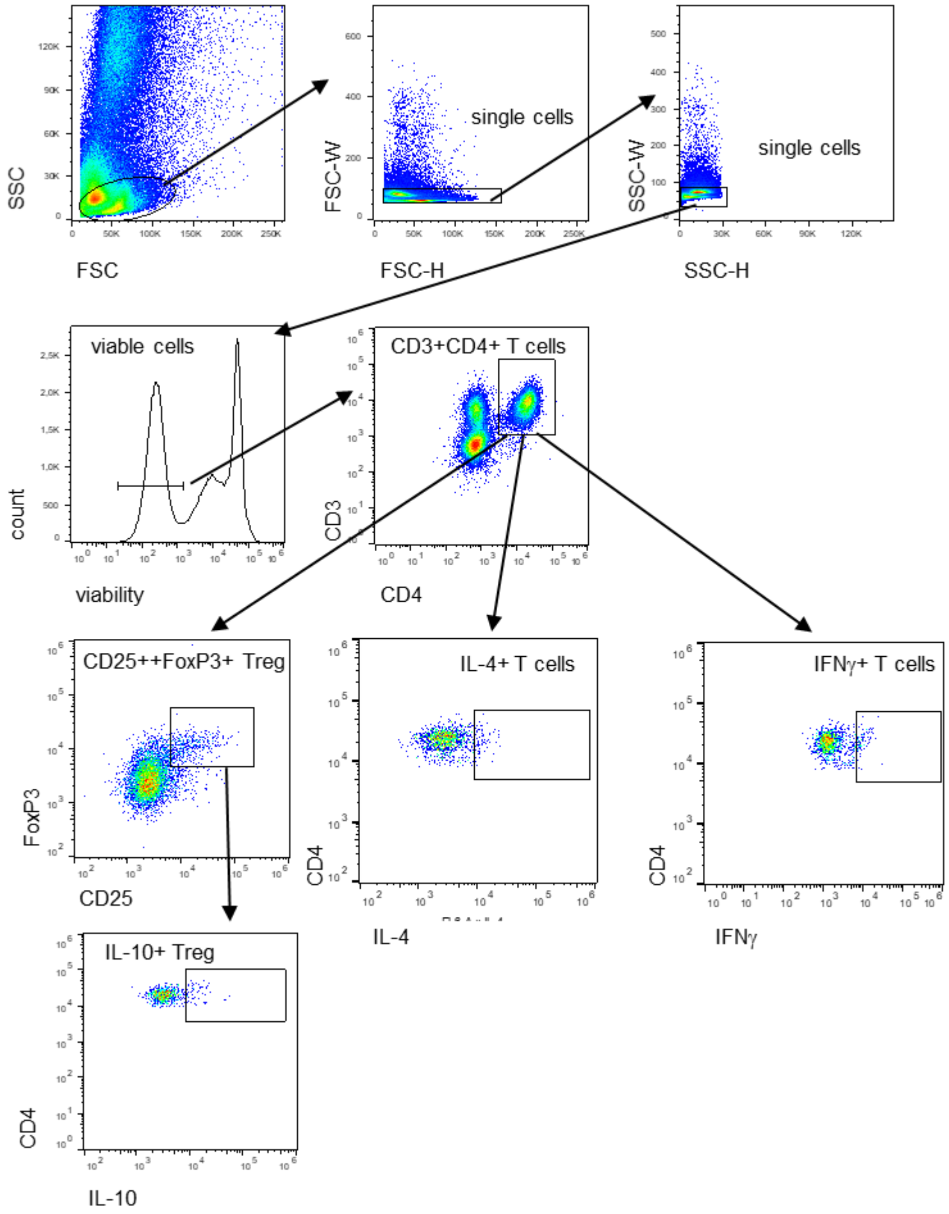

Supplementary Figure 11. Gating strategy for murine lymphocytes.

Gating strategy to quantify intracellular cytokine levels in lung tissue-derived lymphocytes presented in Fig. 6d.

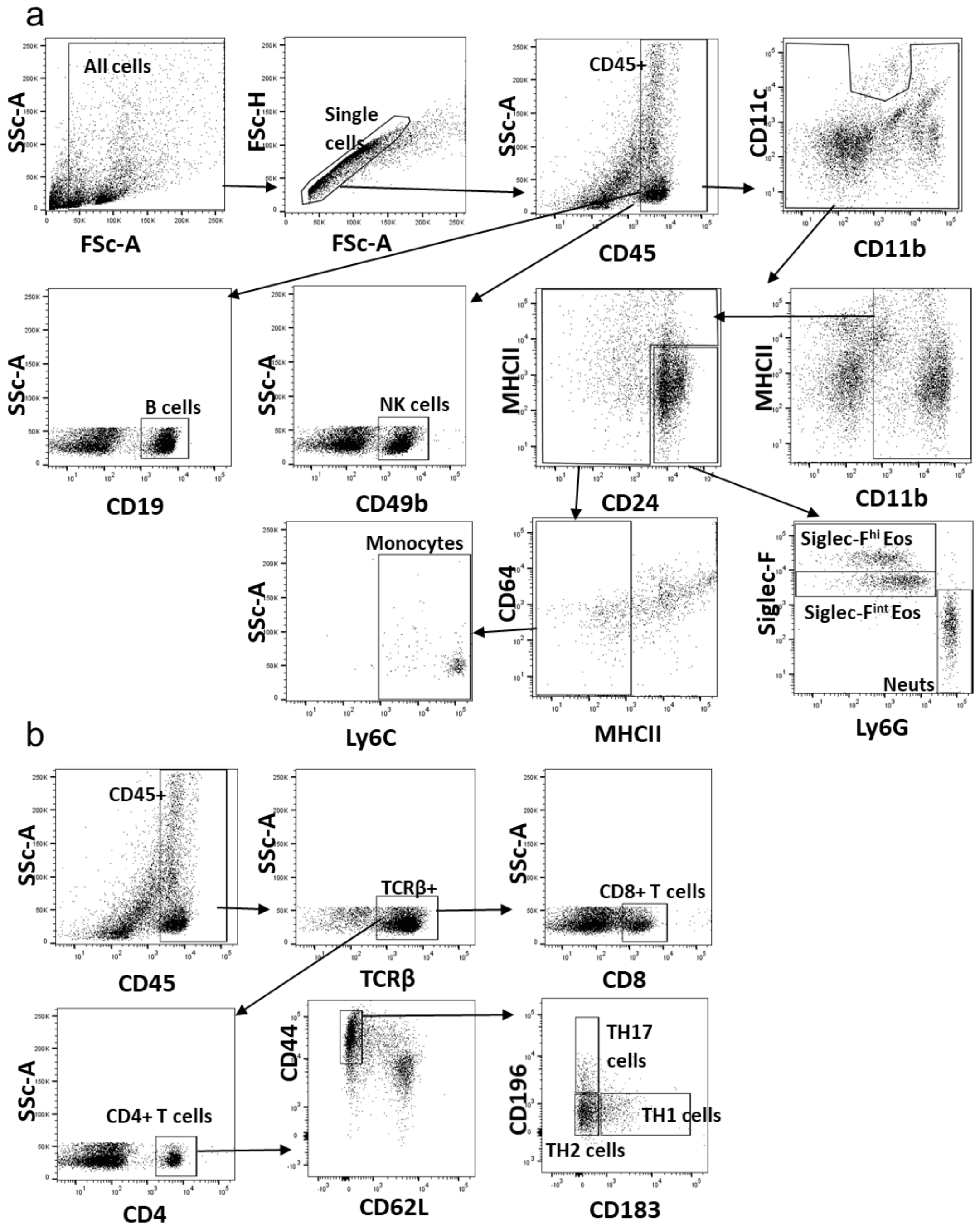

Supplementary Figure 12. Gating strategy for BAL inflammatory cells.

Gating strategy to quantify **a** eosinophils (Eos), neutrophils (Neuts), NK cells, monocytes, B cells and **b** T cell subsets within the murine lung presented in Fig. 7b.

Supplementary Table 1. Patient Comorbidities

	Healthy Controls	Non-Obese Asthma	Obese Non-Asthma	Obese Asthma	p-value
Cardiovascular (%)	2 (4)	6 (11)	22 (43)	20 (40)	< 0.001
Musculoskeletal (%)	2 (4)	0	9 (18)	11 (22)	< 0.001
Gastrointestinal[1] (%)	1 (2)	3 (6)	11 (22)	9 (18)	0.005
Diabetes (%)	0	0	6 (12)	3 (6)	0.010
Neuropsychiatric[2] (%)	0	1 (2)	0	7 (14)	< 0.001

[1]GERD

[2]Depression

Supplementary Table 2. Changes in Microbiome Composition

Genus	Change	Site	Group	p-value	Relative Abundance (Mean)			
					Non-Obese Non-Asthma	Non-Obese Asthma	Obese Non-Asthma	Obese Asthma
Dehalobacterium	Increased	BAL	Asthma[1]	0.007	5.8×10^{-3}	9.4×10^{-3}	2.6×10^{-3}	9.5×10^{-3}
Moraxella	Increased	BAL	Obesity[2]	0.016	9.0×10^{-4}	9.8×10^{-5}	2.6×10^{-3}	5.9×10^{-4}
Atopobium	Increased	BAL	Obesity	0.037	7.1×10^{-5}	9.4×10^{-5}	4.6×10^{-4}	8.9×10^{-4}
Actinomyces	Increased	BAL	Obesity	0.044	1.6×10^{-3}	1.8×10^{-3}	6.3×10^{-3}	9.1×10^{-3}
Veillonella	Increased	BAL	Obesity	0.045	5.1×10^{-2}	2.7×10^{-2}	9.4×10^{-2}	0.159
Dehalobacterium	Increased	Fecal	Asthma	0.032	3.4×10^{-4}	6.1×10^{-4}	2.5×10^{-4}	4.5×10^{-4}
Salmonella	Increased	Fecal	Asthma	0.048	5.7×10^{-6}	3.0×10^{-5}	1.9×10^{-5}	3.6×10^{-5}
Bilophila	Increased	Fecal	Non-Obese Asthma	0.036	4.1×10^{-3}	6.5×10^{-3}	3.3×10^{-3}	4.6×10^{-3}
Anaerovibrio	Increased	Fecal	Obese Non-Asthma	0.011	1.1×10^{-5}	2.0×10^{-5}	6.6×10^{-5}	3.4×10^{-5}
Atopobium	Increased	Fecal	Obese Non-Asthma	0.037	2.0×10^{-5}	1.8×10^{-5}	3.6×10^{-5}	1.9×10^{-5}
Tissierellaceae;g_1-68	Increased	Fecal	Obese Asthma	0.037	4.8×10^{-6}	5.5×10^{-5}	8.2×10^{-6}	6.9×10^{-5}
Flavobacteriaceae;g_unknown	Increased	Fecal	Obesity	0.012	3.1×10^{-5}	4.6×10^{-5}	8.6×10^{-5}	4.9×10^{-5}
Caulobacteraceae;g_unknown	Increased	Fecal	Obesity	0.035	1.3×10^{-5}	1.5×10^{-5}	3.1×10^{-5}	2.1×10^{-5}
Pseudomonas	Increased	Fecal	Obesity	0.039	1.6×10^{-5}	1.2×10^{-5}	2.8×10^{-5}	2.5×10^{-5}
Dehalobacterium	Increased	Nasal	Asthma	0.044	1.3×10^{-6}	6.7×10^{-5}	2.9×10^{-5}	3.8×10^{-3}
Shewanella	Increased	Nasal	Obesity	0.043	1.1×10^{-6}	5.8×10^{-6}	2.2×10^{-5}	8.9×10^{-5}
Peptoniphilus	Increased	Nasal	Obesity	0.046	8.2×10^{-3}	8.3×10^{-3}	2.1×10^{-2}	4.0×10^{-2}
Carnobacteriaceae;g_unknown	Increased	Oral	Obese Non-Asthma	0.047	3.1×10^{-6}	4.4×10^{-6}	4.9×10^{-6}	4.0×10^{-6}
Vagococcus	Increased	Oral	Obesity	0.020	8.7×10^{-6}	2.2×10^{-5}	3.2×10^{-5}	8.8×10^{-5}
Dehalobacteriaceae;g_unknown	Increased	Oral	Obesity	0.025	1.1×10^{-6}	3.2×10^{-6}	2.2×10^{-5}	4.8×10^{-5}
Granulicatella	Increased	Oral	Obesity	0.044	8.5×10^{-3}	1.3×10^{-2}	1.7×10^{-2}	1.5×10^{-2}
Moraxella	Decreased	BAL	Asthma	0.007	9.0×10^{-4}	9.8×10^{-5}	2.6×10^{-3}	5.9×10^{-4}
Bacteroides	Decreased	BAL	Asthma	0.008	4.3×10^{-2}	2.7×10^{-2}	5.3×10^{-2}	1.1×10^{-2}
Coriobacteriaceae;g_unknown	Decreased	BAL	Asthma	0.014	1.1×10^{-3}	2.3×10^{-4}	5.4×10^{-4}	9.3×10^{-5}
Butyricimonas	Decreased	BAL	Asthma	0.018	1.4×10^{-4}	2.7×10^{-5}	7.3×10^{-4}	9.1×10^{-6}
Parabacteroides	Decreased	BAL	Asthma	0.029	3.3×10^{-3}	1.1×10^{-3}	3.6×10^{-3}	2.3×10^{-4}

Bifidobacterium	Decreased	BAL	Asthma	0.029	2.4×10^{-3}	1.2×10^{-3}	2.1×10^{-3}	8.5×10^{-4}
Enterococcus	Decreased	BAL	Asthma	0.030	2.8×10^{-3}	1.4×10^{-3}	2.3×10^{-3}	1.3×10^{-3}
Faecalibacterium	Decreased	BAL	Asthma	0.032	1.5×10^{-3}	5.1×10^{-4}	2.0×10^{-3}	3.6×10^{-5}
Ruminococcaceae;g_unknown	Decreased	BAL	Asthma	0.042	2.7×10^{-2}	2.0×10^{-2}	2.8×10^{-2}	1.4×10^{-2}
Roseburia	Decreased	BAL	Asthma	0.044	1.6×10^{-3}	8.2×10^{-4}	1.8×10^{-3}	1.3×10^{-4}
Propionibacterium	Decreased	BAL	Non-Obese Asthma	0.021	2.5×10^{-5}	0.0	1.2×10^{-4}	3.7×10^{-5}
Campylobacter	Decreased	BAL	Non-Obese Asthma	0.030	9.0×10^{-4}	1.6×10^{-4}	6.7×10^{-4}	5.9×10^{-4}
Mitsuokella	Decreased	BAL	Non-Obese Asthma	0.036	9.3×10^{-5}	3.6×10^{-5}	1.6×10^{-6}	9.4×10^{-5}
Pasteurellaceae;g_unknown	Decreased	BAL	Non-Obese Asthma	0.044	2.1×10^{-5}	6.9×10^{-7}	1.7×10^{-5}	1.8×10^{-5}
Enterococcaceae;g_unknown	Decreased	BAL	Obese Asthma	0.002	2.2×10^{-5}	2.8×10^{-4}	1.8×10^{-5}	2.8×10^{-6}
Phascolarctobacterium	Decreased	BAL	Obese Asthma	0.007	2.0×10^{-2}	2.3×10^{-2}	2.5×10^{-2}	6.9×10^{-3}
Paraprevotella	Decreased	BAL	Obese Asthma	0.012	1.2×10^{-2}	1.1×10^{-2}	1.4×10^{-2}	2.9×10^{-3}
Aeromonadaceae;g_unknown	Decreased	BAL	Obese Asthma	0.039	3.3×10^{-6}	1.6×10^{-6}	1.9×10^{-6}	2.6×10^{-7}
Megasphaera	Decreased	BAL	Obese Asthma	0.023	2.0×10^{-2}	1.1×10^{-2}	8.8×10^{-3}	1.5×10^{-3}
Mitsuokella	Decreased	BAL	Obese Non-Asthma	0.022	9.3×10^{-5}	3.6×10^{-5}	1.6×10^{-6}	9.4×10^{-5}
Ruminococcus	Decreased	BAL	Obese Non-Asthma	0.027	1.2×10^{-3}	1.2×10^{-3}	4.0×10^{-4}	8.9×10^{-4}
Comamonadaceae;g_unknown	Decreased	BAL	Obese Non-Asthma	0.030	1.3×10^{-4}	1.5×10^{-4}	4.0×10^{-5}	8.9×10^{-5}
Dehalobacterium	Decreased	BAL	Obese Non-Asthma	0.034	5.8×10^{-3}	9.4×10^{-3}	2.6×10^{-3}	9.5×10^{-3}
Anaeroplasma	Decreased	BAL	Obesity	0.008	8.6×10^{-3}	1.2×10^{-2}	3.8×10^{-3}	4.5×10^{-3}
Acidaminococcus	Decreased	Fecal	Non-Obese Asthma	0.040	3.0×10^{-2}	2.4×10^{-3}	6.6×10^{-3}	9.1×10^{-3}
Mitsuokella	Decreased	Fecal	Non-Obese Asthma	0.048	3.9×10^{-4}	2.5×10^{-4}	4.8×10^{-4}	3.9×10^{-4}
Serratia	Decreased	Fecal	Obese Asthma	0.044	5.8×10^{-5}	1.1×10^{-3}	3.6×10^{-5}	1.0×10^{-5}
Alistipes	Decreased	Fecal	Obese Non-Asthma	0.013	1.1×10^{-3}	1.8×10^{-3}	4.7×10^{-4}	1.6×10^{-3}
Oxalobacter	Decreased	Fecal	Obese Non-Asthma	0.034	3.3×10^{-4}	2.8×10^{-4}	1.2×10^{-4}	4.1×10^{-4}
Acidaminococcus	Decreased	Fecal	Obese Non-Asthma	0.042	3.0×10^{-2}	2.4×10^{-3}	6.6×10^{-3}	9.1×10^{-3}
Clostridiales;g_unknown	Decreased	Fecal	Obesity	0.003	4.1×10^{-2}	3.7×10^{-2}	1.9×10^{-2}	2.4×10^{-2}
Rothia	Decreased	Fecal	Obesity	0.002	7.8×10^{-5}	6.0×10^{-5}	2.0×10^{-5}	3.5×10^{-5}

Oscillospira	Decreased	Fecal	Obesity	0.004	$1.8x10^{-2}$	$2.0x10^{-2}$	$8.7x10^{-3}$	$1.5x10^{-2}$
Butyricimonas	Decreased	Fecal	Obesity	0.021	$3.4x10^{-3}$	$2.6x10^{-3}$	$1.6x10^{-3}$	$2.1x10^{-3}$
Holdemania	Decreased	Fecal	Obesity	0.026	$1.2x10^{-4}$	$7.8x10^{-5}$	$2.6x10^{-5}$	$3.6x10^{-5}$
Delftia	Decreased	Nasal	Non-Obese Asthma	0.014	$7.5x10^{-5}$	$2.1x10^{-5}$	$4.5x10^{-6}$	$3.7x10^{-5}$
Methylobacteriaceae;g_unknown	Decreased	Nasal	Non-Obese Asthma	0.038	$4.4x10^{-4}$	$1.1x10^{-6}$	$1.2x10^{-4}$	$3.4x10^{-4}$
Rikenellaceae;g_unknown	Decreased	Nasal	Obese Asthma	0.007	$2.4x10^{-5}$	$1.6x10^{-4}$	$4.8x10^{-6}$	$1.2x10^{-6}$
Corynebacterium	Decreased	Nasal	Obese Asthma	0.046	$3.5x10^{-1}$	$2.7x10^{-1}$	$2.8x10^{-1}$	$2.2x10^{-1}$
Delftia	Decreased	Nasal	Obese Non-Asthma	0.004	$7.5x10^{-5}$	$2.1x10^{-5}$	$4.5x10^{-6}$	$3.7x10^{-5}$
Rikenellaceae;g_unknown	Decreased	Nasal	Obese Non-Asthma	0.023	$2.4x10^{-5}$	$1.6x10^{-4}$	$4.8x10^{-6}$	$1.2x10^{-6}$
Methylobacteriaceae;g_unknown	Decreased	Nasal	Obese Non-Asthma	0.045	$4.4x10^{-4}$	$1.1x10^{-6}$	$1.2x10^{-4}$	$3.4x10^{-4}$
Streptophyta;g_unknown	Decreased	Nasal	Obesity	0.028	$2.8x10^{-3}$	$2.4x10^{-3}$	$5.3x10^{-4}$	$4.7x10^{-4}$
Rhodococcus	Decreased	Nasal	Obesity	0.043	$1.1x10^{-4}$	$1.6x10^{-4}$	$6.7x10^{-6}$	$2.6x10^{-5}$
Micrococcaceae;g_unknown	Decreased	Oral	Asthma	0.030	$4.1x10^{-5}$	$1.5x10^{-5}$	$3.1x10^{-5}$	$1.1x10^{-6}$
Peptostreptococcus	Decreased	Oral	Asthma	0.044	$2.1x10^{-3}$	$9.6x10^{-4}$	$2.5x10^{-3}$	$1.0x10^{-3}$
Paraprevotellaceae;g_unknown	Decreased	Oral	Non-Obese Asthma	0.019	$9.8x10^{-5}$	$1.9x10^{-5}$	$5.9x10^{-5}$	$4.1x10^{-5}$
RF32;g_unknown	Decreased	Oral	Non-Obese Asthma	0.034	$3.9x10^{-5}$	$1.3x10^{-6}$	$6.4x10^{-4}$	$1.5x10^{-5}$
Coriobacteriaceae;g_unknown	Decreased	Oral	Non-Obese Asthma	0.035	$8.6x10^{-4}$	$2.0x10^{-4}$	$3.5x10^{-4}$	$7.5x10^{-4}$
Corynebacterium	Decreased	Oral	Non-Obese Asthma	0.041	$3.8x10^{-3}$	$1.6x10^{-3}$	$2.2x10^{-3}$	$3.4x10^{-3}$
Aggregatibacter	Decreased	Oral	Non-Obese Asthma	0.047	$7.0x10^{-3}$	$3.4x10^{-3}$	$6.1x10^{-3}$	$4.4x10^{-3}$
Leptotrichia	Decreased	Oral	Obese Asthma	0.013	$1.6x10^{-2}$	$1.3x10^{-2}$	$1.2x10^{-2}$	$1.1x10^{-2}$
Actinomycetaceae;g_unknown	Decreased	Oral	Obese Asthma	0.020	$7.2x10^{-4}$	$4.6x10^{-4}$	$1.9x10^{-4}$	$1.7x10^{-4}$
Parvimonas	Decreased	Oral	Obese Asthma	0.035	$3.0x10^{-3}$	$1.8x10^{-3}$	$1.9x10^{-3}$	$5.9x10^{-4}$
Schwartzia	Decreased	Oral	Obese Non-Asthma	0.013	$7.0x10^{-4}$	$5.6x10^{-4}$	$2.9x10^{-4}$	$7.8x10^{-4}$
Neisseriaceae;g_unknown	Decreased	Oral	Obese Non-Asthma	0.020	$2.3x10^{-3}$	$1.3x10^{-3}$	$4.4x10^{-4}$	$8.4x10^{-4}$
Christensenellaceae;g_unknown	Decreased	Oral	Obese Non-Asthma	0.037	$2.4x10^{-5}$	$1.7x10^{-4}$	$2.3x10^{-6}$	$6.0x10^{-5}$
Paludibacter	Decreased	Oral	Obese Non-Asthma	0.045	$2.9x10^{-3}$	$1.1x10^{-3}$	$7.9x10^{-4}$	$9.0x10^{-4}$

RF16;g_unknown	Decreased	Oral	Obese Non-Asthma	0.049	$4.6x10^{-6}$	$8.6x10^{-6}$	$5.5x10^{-7}$	$4.3x10^{-5}$
Actinomycetaceae;g_unknown	Decreased	Oral	Obesity	0.012	$7.2x10^{-4}$	$4.6x10^{-4}$	$1.9x10^{-4}$	$1.7x10^{-4}$
GMD14H09;f_;g_unknown	Decreased	Oral	Obesity	0.030	$4.1x10^{-5}$	$3.4x10^{-5}$	$1.1x10^{-6}$	$2.1x10^{-6}$
Staphylococcus	Decreased	Oral	Obesity	0.034	$2.9x10^{-4}$	$9.7x10^{-5}$	$5.1x10^{-5}$	$4.6x10^{-5}$
Peptococcus	Decreased	Oral	Obesity	0.039	$4.4x10^{-4}$	$4.1x10^{-4}$	$1.0x10^{-4}$	$1.1x10^{-4}$

[1]Asthma refers to both obese asthma and non-obese asthma combined; [2]Obesity refers to both obese asthma and obese non-asthma combined.

Supplementary Table 3. Changes in BAL Microbiome Composition Associated with Inflammatory Cell Numbers.

Genus	Cell Type	Association	Group	p-value
Rothia	Eosinophils	Positive	Asthma[1]	0.031
Dorea	Eosinophils	Positive	Asthma	0.027
Lautropia	Eosinophils	Positive	Asthma	0.041
Haemophilus	Eosinophils	Positive	Asthma	0.035
Prevotella	Eosinophils	Positive	Non-Obese Asthma	0.020
[Paraprevotellaceae];g__unknown	Eosinophils	Negative	Non-Obese Asthma	0.012
Clostridiales;g__unknown	Eosinophils	Negative	Non-Obese Asthma	0.019
Lactobacillales;g__unknown	Eosinophils	Negative	Non-Obese Asthma	0.041
RF39;g__unknown	Neutrophils	Positive	Asthma	0.046
[Acidaminobacteraceae];g__unknown	Neutrophils	Positive	Asthma	0.004
Comamonadaceae;g__unknown	Neutrophils	Negative	Asthma	0.017
Ralstonia	Neutrophils	Negative	Asthma	0.045

[1]Asthma refers to both obese asthma and non-obese asthma combined
